# A Distinct Tobamovirus Associated With *Trichosanthes kirilowii* Mottle Mosaic Disease

**DOI:** 10.3389/fmicb.2022.927230

**Published:** 2022-06-21

**Authors:** Cheng Chen, Min Du, Deliang Peng, Wulun Li, Jingfeng Xu, Xiuling Yang, Xueping Zhou

**Affiliations:** ^1^State Key Laboratory for Biology of Plant Diseases and Insect Pests, Institute of Plant Protection, Chinese Academy of Agricultural Sciences, Beijing, China; ^2^Institute of Plant Protection, Sichuan Academy of Agricultural Sciences, Key Laboratory of Integrated Pest Management on Crops in Southwest, Ministry of Agriculture, Chengdu, China; ^3^Service Center of Qianshan Plant-Products Industry, Qianshan, China; ^4^State Key Laboratory of Rice Biology, Institute of Biotechnology, Zhejiang University, Hangzhou, China

**Keywords:** Trichosanthes mottle mosaic virus, Cucurbitaceae, infectious clone, infectivity, mechanical transmission

## Abstract

*Trichosanthes kirilowii* is one of the most important perennial herbaceous vines that have been used in traditional Chinese medicine. In this study, a novel RNA virus was discovered in *T. kirilowii* plants showing leaf mottling and mosaic symptoms. The complete genome of this virus is 6,524 nucleotides long and encodes four open reading frames which are arranged in a manner typical of tobamoviruses. Phylogenetic analysis based on the complete genome sequence revealed that the virus was clustered into a branch with the tobamoviruses whose natural host are plants belonging to the family Cucurbitaceae. A full-length infectious cDNA clone was then constructed and demonstrated to establish a systemic infection with typical symptoms in *Nicotiana benthamiana, T. kirilowii*, and five other cucurbitaceous crops including *Cucumis melo, C. lanatus, C. sativus, Luffa aegyptiaca*, and *Cucurbita pepo via* agrobacterium-mediated infectivity assays. Further experiments provided evidence that the rod-shaped viral particles derived from the infectious clone could be mechanically transmitted and reproduce indistinguishable symptoms in the tested plants. Taken together, the mottle mosaic disease of *T. kirilowii* is caused by a distinct tobamovirus, for which the name Trichosanthes mottle mosaic virus (TrMMV) is proposed. As the infectious cDNA clone of TrMMV could also infect five other cucurbit crops, this distinct tobamovirus could be a potential threat to other cucurbitaceous crops.

## Introduction

*Trichosanthes kirilowii* Maxim. is a vine in the family Cucurbitaceae that is mainly distributed in eastern and southern Asia, including China, South Korea, and Japan (Yu et al., 2018). It is grown for use in traditional Chinese medicine and is considered to be one of the 50 fundamental herbs in Chinese herbalism (Fei et al., 2004). Almost all parts of *T. kirilowii*, including fruit, pericarp, seed, and root, have pharmacological activity. In clinical treatment, *T. kirilowii* plays an important role in the treatment of thoracic obstruction, angina pectoris, heart failure, myocardial infarction, pulmonary heart disease, and cerebral ischemic diseases (Li and Liu, 2016; Zhu et al., 2017). Pharmacological tests have also demonstrated the pharmacological activities of *T. kirilowii* in anti-tumor, anti-oxidation, neuroprotection, lowering blood lipids, and reducing kidney damage (Lv et al., 2018; Lu et al., 2019; Hou et al., 2020; Ku et al., 2020). However, cultivated *T. kirilowii* plants are constantly attacked by a variety of plant diseases, posing a serious threat to the *T. kirilowii* industry (Li and Zhang, 2007; Zhang et al., 2016).

Plant viruses are obligate intracellular parasites that account for almost half of the emerging plant disease-causing pathogens. *Tobamovirus* is the largest genus in the family *Virgaviridae*, and several viruses belonging to this genus have long been a serious threat to agriculture at a global level. Tobamoviruses encompass 37 species and have caused devastating epidemics and significant yield and economic losses in many economically important crops, especially in those belonging to the Solanaceae and Cucurbitaceae families (Dombrovsky et al., 2017). They are characterized by typical helical rod particles of about 18 × 300 nm and a central hollow core of 4 nm in diameter. The genomes of tobamoviruses consist of a single-stranded positive-sense RNA that is 6,300–6,800 nucleotides (nts) long and encode four open reading frames (ORFs) (Melcher et al., 2021). Two overlapping ORFs start from the same 5’ proximal start codon, one of which terminates at the first in-frame stop codon and encodes a 125–130 kDa protein. The other ORF generates a 180–190 kDa protein about 5–10% of the time by read-through of the leaky termination codon. Both the 125–130 kDa and the 180–190 kDa proteins are replicase and participate in virus replication. The third ORF encodes a 28–34 kDa movement protein (MP) that transports the viral genome to neighboring plant cells through plasmodesmata (Conti et al., 2017). The 3’ proximal ORF encodes a 17–18 kDa coat protein (CP), which is involved in particle assembly, long-distance movement of virus, and symptom development (Melcher et al., 2021).

In this study, a novel tobamovirus was identified from *T. kirilowii* plants showing mottling and mosaic symptoms. A full-length cDNA clone was further developed and demonstrated to be infectious to *T. kirilowii, Nicotiana benthamiana*, and other five different cucurbit plant species. According to the tobamovirus species demarcation criteria set by the International Committee on Taxonomy of Viruses, the name Trichosanthes mottle mosaic virus (TrMMV) is proposed.

## Materials and Methods

### Plant Materials

**FIGURE 1:**
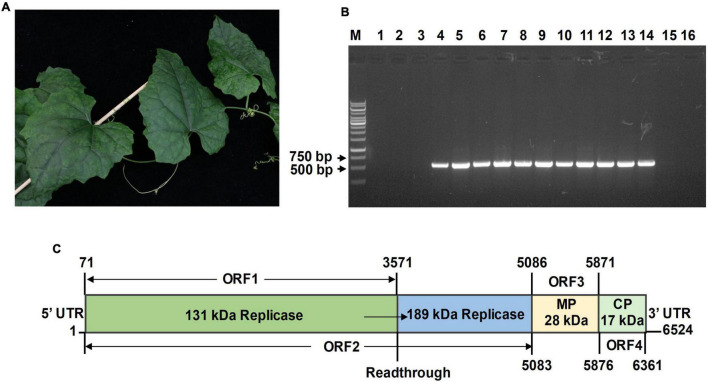
Features of Trichosanthes mottle mosaic virus (TrMMV). **(A)** Leaf mottling and mosaic symptoms associated with the diseased *Trichosanthes kirilowii*. **(B)** Detection of TrMMV by RT-PCR with TrMMV-specific primers (TrMMV-F/TrMMV-R, Supplementary Table 1). Lane M, GeneRuler 1 kb DNA ladders (Thermo); lane 1–14, cDNA extracted from the 14 *T. kirilowii* samples were used as templates for RT-PCR; lane 15 and 16, cDNA extracted from virus-free *T. kirilowii* plant and ddH_2_O were used as the negative controls, respectively. **(C)** Genomic organization of TrMMV. The numbers indicate the position of nucleotides. The protein products encoded by each open reading frame (ORF) are listed. The 189-kDa replicase is encoded by ORF2 *via* a read-through mechanism. MP, movement protein; CP, coat protein.

*T. kirilowii* plants showing leaf mottling and mosaic symptoms (Figure 1A) were collected from Qianshan City, Anhui Province of China in 2021. *T. kirilowii* virus-free seedlings were gifted by Dr. Zhuannan Chu from Anhui Academy of Agricultural Sciences. *Cucumis melo* cv. Shuaiguo 9, *C. lanatus* cv. Ruixin, *C. sativus* cv. Zhongnong 26, *Luffa aegyptiaca* cv. Jiayuantiangesi, and *Cucurbita pepo* cv. Zhonghu 16 seeds were obtained from the Institute of Vegetables and Flowers, Chinese Academy of Agricultural Sciences. The plants were placed in a growth chamber at 25°C with a 16:10-h (light/dark) photoperiod.

### RNA Extraction and Deep Sequencing

Total RNA was extracted from *T. kirilowii* leaves using TRIzol reagent (Invitrogen, Carlsbad, CA, United States) according to the manufacturer’s instructions. The qualified total RNA was used to construct the small RNA (sRNA) library according to the instructions of the Small RNA Sample Pre-Kit (New England BioLabs, Ipswich, MA. United States) (Su et al., 2016). The resulting library was submitted to the Illumina HiSeq 2000 platform for sequencing (Novogene, Beijing China). After sequencing, clean reads were obtained by removing the adapter sequences and low-quality sequences. sRNAs of 18–26 nt were assembled into contigs using the Velvet 0.7.31 software with k-mers of 17 (Zerbino and Birney, 2008). BLASTN of the NCBI GenBank database was used to analyze the assembled contigs to determine the type of candidate virus.

### Genome Assembly and Sequence Analysis

To obtain the full genome sequence of the virus, three primer sets (GL1F/1R, GL2F/2R, and GL3F/3R, Supplementary Table 1) were designed based on the sequences of assembled contigs and used to amplify adjacent regions with overlapping regions. First-strand cDNA was synthesized by reverse transcription using PrimeScript RT Reagent Kit (Takara, Tokyo, Japan) according to the manufacturer’s instructions. The PCR amplification was conducted by TransStart^®^ FastPfu DNA Polymerase (TransGen, Beijing, China), and the amplified products were purified by Gel Extraction Kit (Omega, Norcross, GA, United States) and ligated into pEASY-Blunt vector (TransGen) for sequencing. The exact 5′- and 3′-terminal sequences of viral genomic RNA were determined using the SMARTer^®^ RACE 5′/3′ Kit (Clontech, CA, United States). The sequences of 5′ and 3′ gene-specific primers (5′RACE-GSP1, 3′RACE-GSP2) and nested primers (5′RACE-NGSP3, 3′RACE-NGSP4) are shown in Supplementary Table 1. PCR products were cloned into the pRACE Vector (Takara) and sequenced. Sequences were edited and assembled using the DNAStar 7.01 software (Madison, WI, United States). The assembled complete genome sequence was deposited in the GenBank database as accession number OL404963.

### Virus Genome Sequence Analysis

ORFs encoded by the complete viral genome were predicted using ORF Finder and then manually corrected by comparison with related species deposited in the GenBank database. The similarity analysis of nucleotide and deduced amino acid sequences was conducted by pairwise sequence alignment tools. Phylogenetic analyses based on amino acid sequences were performed by the neighbor-joining method using MEGA X software (Kumar et al., 2018). Phylogenetic trees of the complete genome sequences of viruses were constructed as described (Chen et al., 2020). Briefly, MAFFT v7.037 software (Katoh and Standley, 2013) was used to align the gene sequence, and then SequenceMatrix v1.7.8 software (Lanfear et al., 2017) was used to convert the aligned sequences into NEXUS (non-interleaved) format file. The Bayesian inference (BI) method was used for phylogenetic analysis based on the complete genome sequences with the MrBayes v3.2.6 program (Ronquist et al., 2012).

### Construction of the Infectious cDNA Clone

The infectious cDNA clone of TrMMV was constructed into the plant binary vector pCB301 using a one-step assembly strategy as described by Ma et al. (2021). Briefly, two overlapping DNA fragments were amplified using TrMMV-insert1F/1R, TrMMV-insert2F/2R (Supplementary Table 1), respectively. At the same time, the FastDigest restriction enzymes *Bam*HI and *Stu*I (Thermo Fisher, Waltham, United States) were used for the linearization of the pCB301 vector. After gel purification, the PCR products of the two amplified virus fragments and the linearized pCB301 vector were assembled using a ClonExpress II one-step cloning kit (Vazyme, Nanjing, China) according to the manufacturer’s protocol. The recombinant plasmid pCB301-TrMMV was confirmed by Sanger sequencing.

### Agroinfiltration of *Trichosanthes kirilowii* and Other Herbaceous Plants

The recombinant pCB301-TrMMV binary plasmid was transformed into the *Agrobacterium tumefaciens* strain EHA105 *via* electroporation (Mersereau et al., 1990). *A. tumefaciens*-mediated virus inoculation was carried out as previously described (Yang et al., 2018). After the *A. tumefaciens* cells were cultured and harvested, they were resuspended in a solution containing 10 mM MgCl_2_, 10 mM MES (pH 5.8), and 100 μM acetosyringone with OD600 = 1.0. Approximately 400 μL of *A. tumefaciens* suspension was infiltrated into the abaxial side of the upper two leaves of 21-day-old *T. kirilowii* using a needleless syringe. For agroinoculation of *N. benthamiana* plants, three fully expanded upper leaves of 4-week-old plants were infiltrated. For agroinoculation of cucurbitaceous plants, fully expanded cotyledons were infiltrated with a needleless syringe. Infiltration experiments were conducted at least twice and five plants were used for each treatment. Plants mock-inoculated with *A. tumefaciens* harboring the pCB301 vector served as negative controls. All the inoculated plants were maintained in a growth chamber at 25°C with a 16 h light/10 h dark cycle.

### Mechanical Inoculation

Symptomatic leaves of *N. benthamiana* infiltrated with *A. tumefaciens* harboring pCB301-TrMMV were used as the source of inoculum. Approximately 1.0 g of *N. benthamiana* leaf samples was ground in 10 mL of 0.01 M phosphate buffer (pH 7.2) and used as crude sap (Simkovich et al., 2021). Two leaves of *N. benthamiana, T. kirilowii, C*. *melo, C*. *sativus, C*. *lanatus, L*. *aegyptiaca*, and *C*. *pepo* plants were dusted with carborundum powder and gently rubbed with the ground leaf sap. After 5 min, the inoculated leaves were rinsed with water to eliminate the excess carborundum. Mechanical inoculation tests were conducted at least twice, and five plants were used for each treatment.

### Transmission Electron Microscopy

Transmission electron microscopy was used to observe the virus particles. Approximately 0.1 g of *N. benthaminana* or *T. kirilowii* leaf samples were ground with liquid nitrogen and homogenized in 1 mL of 0.01 M phosphate buffer (pH 7.2). After centrifugation at 11,300 × g for 10 min, the supernatant was placed in a copper grid and a filter paper was used to remove impurities and moisture. The grids were covered with a drop of 2% tungsten phosphate staining solution (pH 7.0) and then dried at room temperature. Grids were observed and photographed with a transmission electron microscope (Hitachi, H-7650) at a voltage of 80 kV.

## Results

### Identification of a Tobamovirus in *Trichosanthes kirilowii* Plants Using Deep Sequencing

In April 2021, *T. kirilowii* plants showing mosaic and mottling symptoms were observed in Qianshan city of Anhui Province, China (Figure 1A). To identify potential virus (es) present in the diseased *T. kirilowii* plants, leaves from 14 randomly collected *T. kirilowii* plant samples were pooled and subjected to small RNA deep sequencing. After filtering the adapter sequences and the low-quality sequences, a total of 11,807,487 clean reads were obtained. 9,584,353 clean reads of length 18–26 nts were further assembled into 598 contigs using the Velvet Assembler 0.7.31 (Zerbino and Birney, 2008). BLASTn searches of the GenBank databases revealed that 80 contigs were related to viruses and 49 of which were found to have top similarities to the genomic sequences of zucchini green mottle mosaic virus (ZGMMV) (accession number MF066176), kyuri green mottle mosaic virus (KGMMV) (accession number AJ295948), and cucumber fruit mottle mosaic virus (CFMMV) (accession number JN226146). The longest contig (245 nts) was closely related to ZGMMV (accession number MF066176), with 98% coverage and 90.08% identity at the nucleotide level, indicating the presence of a tobamovirus infection in the samples. To validate the presence of potential tobamovirus in each *T. kirilowii* sample, a pair of specific primers (TrMMV-F/TrMMV-R, Supplementary Table 1) was designed based on known contigs and reverse transcription-PCR (RT-PCR) was performed using the cDNAs prepared from the collected *T. kirilowii* samples as templates. About 520-bp amplicons were produced from 11 of the 14 tested *T. kirilowii* samples (Figure 1B), indicating that most *T. kirilowii* samples were infected by the virus.

To obtain the full genome sequence of the virus, three RT-PCR reactions were individually carried out to amplify three overlapping fragments using three pairs of primers GL1F/1R, GL2F/2R, and GL3F/3R designed based on the contig sequences (Supplementary Table 1). After sequencing and assembly of the RT-PCR products into a continuous sequence, the 5′-terminus and 3′-terminus of viral genomes were determined by 5′ and 3′ RACE amplification and sequencing. Finally, the complete genome of the virus was determined to have a length of 6,524 nts (Figure 1C). Based on SMART analysis and pairwise comparisons with related tobamoviruses, the genome organization of the virus resembles those of members of the genus *Tobamovirus*, encoding four predicted ORFs. The overlapping ORF1 and ORF2 begin at the same start codon site (ATG, position 71). ORF1 terminates at position 3,571 with UAG as the termination codon and encodes a 131-kDa replicase protein including viral RNA helicase (Pfam E-value:5.68e-42) and viral methyltransferase (Pfam E-value: 4.42e-52). ORF2, which terminates at position 5,083 by reading through the leaky termination codon, encodes a 189-kDa replicase protein that includes viral methyltransferase (Pfam E-value: 2.08e-45), viral RNA helicase (Pfam E-value: 3.86e-52), and RNA-dependent RNA polymerase (Pfam E-value: 3.03e-149) domains. ORF3 and ORF4 encode a 28-kDa MP (Pfam E-value: 4.83e-17) and a 17-kDa CP (Pfam E-value: 2.78e-06), respectively. The 5′ untranslated region (UTR) and 3′ UTR of the virus are 70 and 163 nts in length, respectively (Figure 1C).

### Comparative Genome and Phylogenetic Analysis

BLASTN search using the assembled 6,524-nt viral genome suggested that the viral sequences shared the highest nucleotide identity (87.8%) with ZGMMV-ZT-1 (Accession number AJ252189). Pairwise genome similarity comparison of the tobamovirus to known tobamoviruses showed that the full-length virus genome shared 71.9–87.8% nucleotide similarity with known tobamoviruses (Table 1). According to the species demarcation criteria of the genus *Tobamovirus* (a threshold of 10% nucleotide variability) (Melcher et al., 2021), we propose that this virus belongs to a tentative new species of the genus *Tobamovirus*, which was provisionally named Trichosanthes mottle mosaic virus (TrMMV).

**TABLE 1:** Percentage of nucleotide (nt) and amino acid (aa) sequence identities of Trichosanthes mottle mosaic virus to other nine tobamoviruses.

Virus	Complete sequence	131 kDa replicase		189 kDa replicase		28 kDa (MP)		17 kDa (CP)	
		nt	aa	nt	aa	nt	aa	nt	aa
ZGMMV-ZT-1	87.8%	86.6%	96.0%	87.4%	99.3%	87.4%	94.3%	90.5%	94.4%
ZGMMV	87.6%	86.4%	96.0%	87.3%	99.2%	87.4%	94.3%	90.5%	94.4%
ZGMMV-GXBG1	87.6%	86.4%	96.0%	87.3%	100.0%	87.5%	94.3%	90.5%	94.4%
KGMMV	84.0%	83.4%	93.1%	84.1%	93.8%	84.4%	90.1%	80.7%	77.0%
KGMMV-Yodo	81.7%	81.6%	91.3%	81.8%	91.8%	82.9%	88.2%	80.2%	75.8%
KGMMV-YM	81.4%	81.5%	90.3%	80.9%	87.4%	83.5%	89.7%	79.2%	75.2%
CFMMV-Su12-25	72.8%	72.3%	83.0%	74.0%	83.7%	71.7%	77.1%	74.1%	77.3%
CFMMV-Cm	72.2%	71.6%	82.4%	73.1%	83.4%	70.7%	73.8%	77.4%	78.4%
CFMMV	71.9%	70.9%	80.8%	72.8%	81.4%	70.3%	73.8%	77.2%	77.8%

*Sequences obtained from GenBank: zucchini green mottle mosaic virus (ZGMMV-ZT-1, AJ252189.2; ZGMMV, AJ295949.1; ZGMMV-GXBG1, MF066176.1), kyuri green mottle mosaic virus (KGMMV, AJ295948.1; KGMMV- Yodo, AB015145.1; KGMMV-YM, AB162006.1), and cucumber fruit mottle mosaic virus (CFMMV-Su12-25, MT989352.1; CFMMV-Cm, JN226146.1; CFMMV, AF321057.1).*

Comparison of the nucleotide sequence of ORF1, ORF2, ORF3, and ORF4 of TrMMV to those of other tobamoviruses revealed that TrMMV is most closely relative to ZGMMV-ZT-1. The four ORFs of TrMMV shared nucleotide sequence identity of 86.6, 87.4, 87.4, and 90.5% to those of ZGMMV-ZT-1, respectively (Table 1). The amino acid identities of the four ORFs of TrMMV and ZGMMV-ZT-1 ranged from 94.3 to 99.3% (Table 1).

**FIGURE 2:**
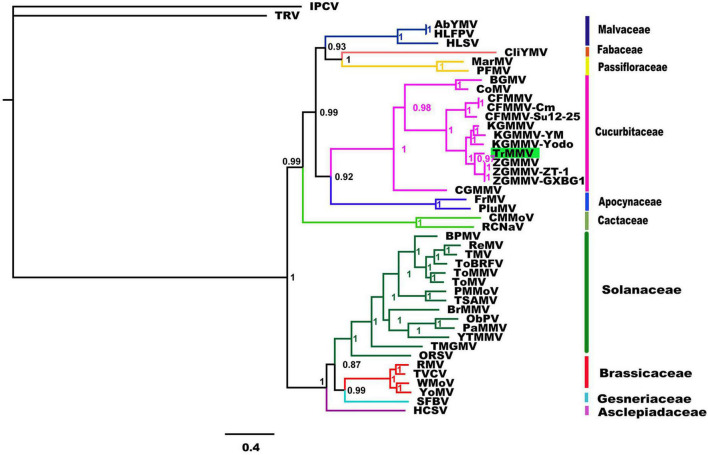
Bayesian phylogenies of whole-genome nucleotide sequences of 43 tobamoviruses. The whole-genome nucleotide sequences of tobacco rattle virus (TRV) and Indian peanut clump virus (IPTV) were used as the outgroups. Support values are Bayesian posterior probabilities. Species and the NCBI accession number used for phylogenetic analyses are provided in Supplementary Table 2.

To better understand the relationship of TrMMV to other known tobamoviruses, a phylogenetic tree was constructed based on the complete genomes of TrMMV and 43 species of the genus *Tobamovirus* using the complete genomes of tobacco rattle virus and Indian peanut clump virus as outgroups. As shown in Figure 2, the 43 representative tobamoviruses could be divided into four groups, mainly corresponding to their natural hosts (Malvaceae, Fabaceae, and Passifloraceae), (Cucurbitaceae and Apocynaceae), Cactaceae, and (Solanaceae, Brassicaceae, Gesneriaceae, and Asclepiadaceae). TrMMV was clustered into a branch with the viruses whose natural host is a plant of the family Cucurbitaceae. Phylogenetic trees constructed based on the deduced amino acid sequences of four ORFs of TrMMV and representative tobamoviruses revealed that the products of ORF1, ORF3, and ORF4 of TrMMV are individually clustered in a separate branch with the ORF1, ORF3, and ORF4 of other tobamoviruses (Supplementary Figure 1), which showed similar topologies to the whole genome sequence.

### Pathogenicity of the Infectious Clone of *Trichosanthes* Mottle Mosaic Virus to *Nicotiana benthamiana*

**FIGURE 3:**
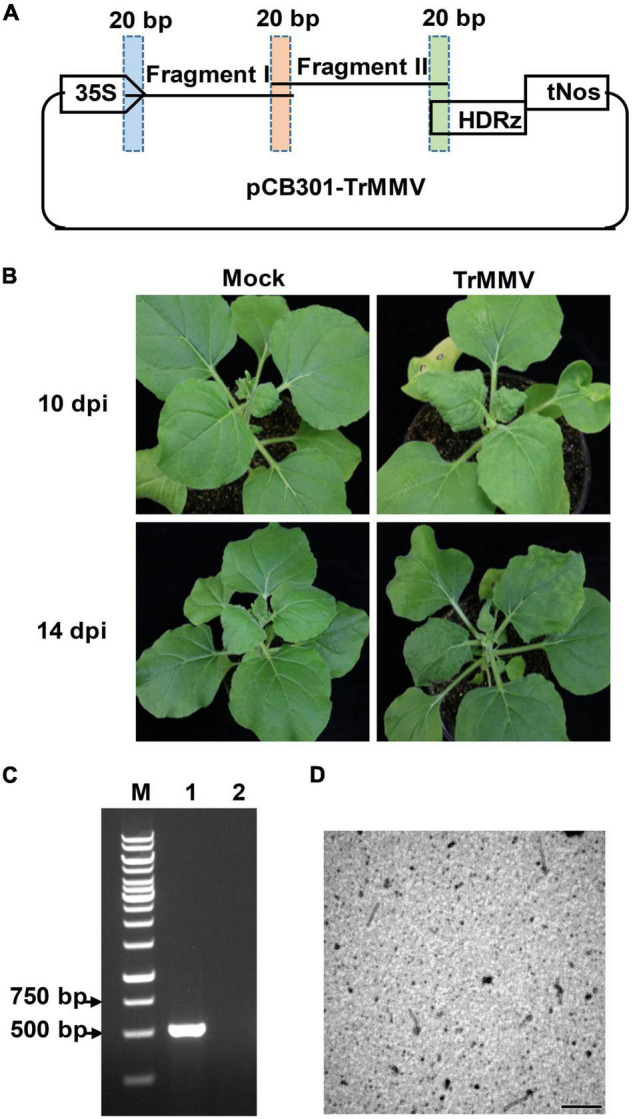
Infectivity of TrMMV on *Nicotiana benthamiana*. **(A)** Schematic diagram illustrating the one-step assembly cloning strategy used for the construction of the infectious full-length cDNA clone of TrMMV. Two overlapping TrMMV fragments, I and II, were amplified by polymerase chain reaction and infused to the linearized pCB301 vector. 35S, the cauliflower mosaic virus promoter; HDRz, hepatitis delta virus ribozyme; tNos, Nos terminator. **(B)** Leaf curling and chlorosis symptoms of *N. benthamiana* infected by the TrMMV infectious clone at 14 days post infiltration (dpi) and 17 dpi, respectively. **(C)** RT-PCR analysis of TrMMV RNA accumulation in systemic leaves of inoculated *N. benthamiana* using TrMMV-specific primers. Lane M, GeneRuler 1kb DNA ladders (Thermo); lane 1, *N. benthamiana* plants agroinoculated with pCB301-TrMMV at 14 dpi; lane 2, mock plants inoculated with the empty pCB301 vector. **(D)** Transmission electron micrographs of viral particles in systemic leaves of *N. benthamiana* plants agroinoculated with pCB301-TrMMV. Bar = 500 nm.

To obtain an infectious clone suitable for agroinfection, the full-length cDNA of TrMMV was cloned into the binary vector pCB301 between the transcription regulatory elements (the cauliflower mosaic virus 35S promoter and the hepatitis delta virus ribozyme) to yield pCB301-TrMMV (Figure 3A). The infectivity of the infectious cDNA clone of TrMMV was primarily tested in the model plant *N. benthamiana* using agrobacterium-mediated infiltration assays. At 10 days post-inoculation (dpi), typical symptoms such as leaf curling and chlorosis began to appear in the non-inoculated systemic leaves of all the 15 inoculated *N. benthamiana* plants (Figure 3B). The presence of the virus on the upper leaves could be detected by RT-PCR (Figure 3C). In contrast, the mock plants inoculated with the empty pCB301 vector showed no obvious disease symptoms and no accumulation of viral RNA (Figure 3C). Electron microscopy identified filamentous particles of 270–320 nm long and 15–20 nm wide in the symptomatic leaf tissues of *N. benthamiana* inoculated with pCB301-TrMMV, representing typical tobamovirus virions (Figure 3D). These results suggested that the TrMMV full-length cDNA clone could replicate and move in *N. benthamiana* plants.

### Pathogenicity of *Trichosanthes* Mottle Mosaic Virus to *Trichosanthes kirilowii*

**FIGURE 4:**
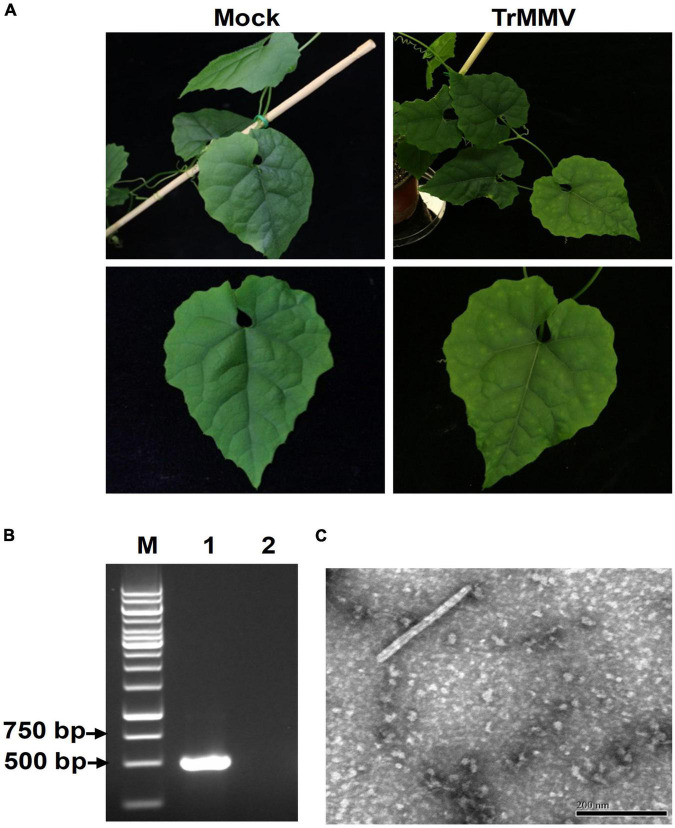
Infectivity of TrMMV on *Trichosanthes kirilowii.* **(A)** Leaf mottling and mosaic symptoms of *T. kirilowii* infected by the TrMMV infectious clone at 17 dpi. **(B)** RT-PCR analysis of TrMMV RNA accumulation in systemic leaves of inoculated *T. kirilowii* using TrMMV-specific primers. Lane M, GeneRuler 1-kb DNA ladders (Thermo); lane 1, *T. kirilowii* plants agroinoculated with pCB301-TrMMV at 17 dpi; lane 2, mock plants inoculated with empty pCB301 vectors. **(C)** The typical rod-shaped virion in systemic leaves of *T. kirilowii* plants agroinoculated with pCB301-TrMMV. Bar = 200 nm.

To evaluate the infectivity of the infectious TrMMV clone on natural hosts, pCB301-TrMMV was agroinfiltrated into virus-free seedlings of *T. kirilowii.* At 17 dpi, typical symptoms such as leaf mosaic and mottling symptoms appeared in the non-inoculated systemic leaves of all the 10 inoculated *T. kirilowii* plants (Figure 4A). RT-PCR detection confirmed the presence of viral RNA in the systemic leaves of inoculated *T. kirilowii* (Figure 4B). In contrast, the mock plants inoculated with the empty pCB301 vector showed no obvious disease symptoms and no accumulation of viral RNA (Figures 4A,B). When crude sap was extracted from the uninoculated symptomatic leaves of *T. kirilowii* plants, typical tobamovirus particles were visible by electron microscopy (Figure 4C).

### Pathogenicity of *Trichosanthes* Mottle Mosaic Virus to Other Cucurbitaceous Crops

**FIGURE 5:**
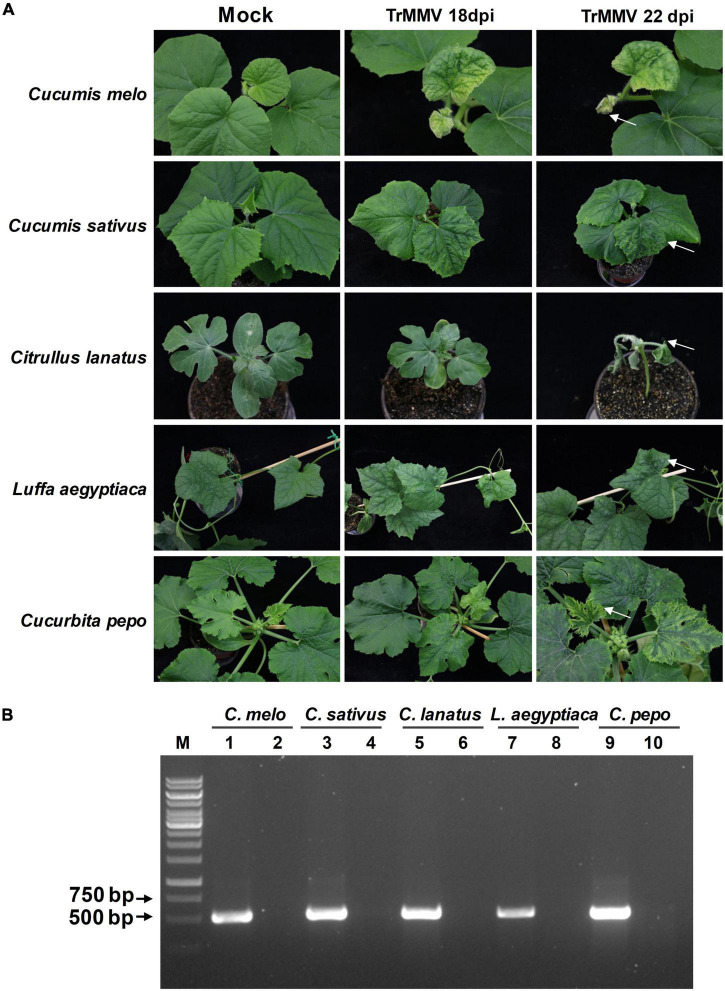
Infectivity of TrMMV on five common cucurbitaceous crops. **(A)** Typical virus infection symptoms of *Cucumis melo, C. sativus, C. lanatus, Luffa aegyptiaca*, and *Cucurbita pepo* plants inoculated with pCB301-TrMMV at 18 and 22 dpi, respectively. Arrows indicate that TrMMV causes the symptoms of systematic leaf necrosis in *C. melo*, leaf crinkle in *C. sativus*, wilting in *C. lanatus* plant, leaf crinkle in *L. aegyptiaca*, and leaf edge chlorosis in *C. pepo*. **(B)** RT-PCR detection of TrMMV in systemic leaves of inoculated plants using TrMMV-specific primers. Lane M, GeneRuler 1kb DNA ladders (Thermo); lane 1, 3, 5, 7, 9, samples collected from systemic leaves of inoculated *C. melo, C. sativus, C. lanatus, L. aegyptiaca*, and *C. pepo* plants, respectively; lane 2, 4, 6, 8, 10, mock plants inoculated with the empty pCB301 vector (*C. melo, C. sativus, C. lanatus, L. aegyptiaca*, and *C. pepo* plants, respectively).

To determine the infectivity of TrMMV to common cucurbitaceous crops, the infectious clone of TrMMV was used to infiltrate the other five cucurbitaceous crops (*C. melo, C. sativus, C. lanatus, L. aegyptiaca*, and *C. pepo*). At 18 dpi, the systemic leaves of *C. melo* seedlings showed symptoms of yellowing, crinkle, and chlorosis (Figure 5A). With the spread of the virus, the systemic leaves of *C. melo* were necrotic from the edge (Figure 5A). For *C. sativus* seedlings infected with TrMMV, the uninoculated systematic leaves showed the symptoms of mottle, chlorosis, and crinkle (Figure 5A). *C. lanatus* inoculated with TrMMV initially showed symptoms of mosaic and chlorosis, followed by whole plant wilting and necrosis (Figure 5A). The systematic leaves of TrMMV-inoculated *L. aegyptiaca* showed symptoms of crinkle and mosaic, which extended inward from the edge of the leaves (Figure 5A). For *C. pepo* inoculated with TrMMV, the systemic leaves showed chlorosis and mosaic symptoms, especially chlorosis at the edge of systematic leaves in the late stage (Figure 5A). To confirm that these symptoms were caused by TrMMV infection, symptomatic and mock samples were tested using RT-PCR, and symptomatic plants were indeed positive for TrMMV (Figure 5B). It is noteworthy that the infection efficacy of the infectious cDNA clone of TrMMV also reached 100% in the tested five cucurbitaceous crops, suggesting that TrMMV is highly infectious to the tested cucurbitaceous crops.

### *Trichosanthes* Mottle Mosaic Virus Derived From the Infectious Clone Is Mechanically Transmissible

**FIGURE 6:**
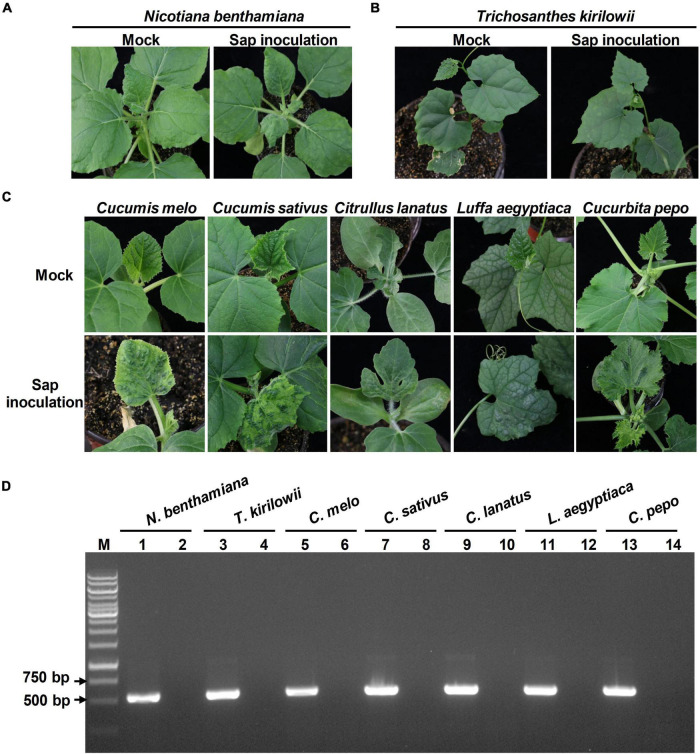
Pathogenicity of TrMMV to *Nicotiana benthamiana, Trichosanthes kirilowii*, and five cucurbitaceous crops based on mechanical inoculation. TrMMV-infected *N. benthamiana* plants (previously agroinfiltrated with the TrMMV infectious clone) were used as inoculum for sap inoculation. Mock plants were rub-inoculated with PBS buffer. **(A)** Mechanical inoculation phenotype of *N. benthamiana.* **(B)** Phenotypes of *T. kirilowii* at 10 days after mechanical inoculation. **(C)** Phenotypes of *Cucumis melo, C. sativus, C. lanatus, Luffa aegyptiaca*, and *Cucurbita pepo* plants infected by sap inoculation at 12 dpi. **(D)** RT-PCR detection of TrMMV in systemic leaves of rub-inoculated plants using TrMMV-specific primers. Lane M, GeneRuler 1 kb DNA ladders (Thermo); lane 1, 3, 5, 7, 9, 11, 13, samples collected from systemic leaves of mechanically inoculated *N. benthamiana, T. kirilowii, C. melo, C. sativus, C. lanatus, L. aegyptiaca*, and *C. pepo* plants, respectively; lanes 2, 4, 6, 8, 10, 12, 14, samples mock-inoculated with PBS buffer.

Previous studies showed that tobamoviruses could be spread rapidly by mechanical contacts. To evaluate whether the viral progeny produced by the infectious TrMMV clone is mechanically transmissible, the *N. benthamiana* leaves agroinfiltrated with the infectious clone of TrMMV were harvested at 14 dpi and rubbed onto the leaves of healthy *N*. *benthamiana, T*. *kirilowii, C*. *melo, C*. *sativus, C*. *lanatus, L*. *aegyptiaca*, and *C*. *pepo* plants, respectively. The symptoms displayed on the systematic leaves of each sap-inoculated plant species showed no significant difference from those inoculated with the infectious clone of TrMMV (Figures 6A–C). RT-PCR detection confirmed that viral RNA was present in the systemic leaves of sap-inoculated plants (Figure 6D). These results indicated that viral progeny derived from the infectious TrMMV clone is biologically active and can be transmitted mechanically.

## Discussion

*T*. *kirilowii* is commercially grown in China because of its medicinal value and economic benefits. Previous studies showed that cucurbit mild mosaic virus can infect *T. kirilowii* and produce mild mosaic symptoms on the upper leaves and bright yellow color on the lower leaves (Fan et al., 2013). A new Trichosanthes associated rhabdovirus 1 was also identified by analyzing a transcriptome dataset of a root sample of *T. kirilowii* (Goh et al., 2020). In this study, we characterized a novel tobamovirus from *T. kirilowii* using sRNA deep sequencing and RT-PCR, for which a tentative name “Trichosanthes mottle mosaic virus (TrMMV)” is proposed. Koch’s postulates assay demonstrated that TrMMV is the causal agent associated with the *T. kirilowii* mottle mosaic disease.

The complete genome of TrMMV shares the highest nucleotide sequence identity (87.8%) with ZGMMV and has a genome organization resembling those of other known tobamoviruses (Melcher et al., 2021). Sequence analysis of the four individual ORFs of TrMMV revealed that the nucleotide sequence identity between the four ORFs encoded by TrMMV and the closely related ZGMMV-ZT-1 ranged from 86.6 to 90.5% (Table 1). Phylogenetic analysis conducted using the complete nucleotide sequences and the deduced amino acid sequences of ORF1, ORF3, and ORF4 revealed that TrMMV was grouped into a single branch in the evolutionary tree, suggesting that TrMMV is a distinct tobamovirus (Melcher et al., 2021). Intriguingly, the amino acid identities of the four ORFs encoded by TrMMV and ZGMMV reached 94.3–99.3%. High amino acid identities of different ORFs have also been observed in tobamoviruses belonging to different species. For example, the amino acid sequences of ORF1, ORF2, and ORF4 of ribgrass mosaic virus (accession number HQ667979) shares 94.2, 94.4, and 91.7% with those of turnip vein-clearing virus (accession number U03387), respectively. The high similarity of amino acid sequences encoded by proteins among different species is due to the degeneracy of codons (Novoa et al., 2019). Host adaptation is a major factor in the sequence convergence or divergence of viruses (Phan et al., 2014). Previous phylogeny studies using 185 full-length genome sequences representing 29 tobamovirus species indicated that tobamoviruses have probably co-diverged with their eudicotyledonous hosts in a “fuzzy” way (Gibbs et al., 2015). It would be interesting to know whether host switches drive the genetic variation between TrMMV and other tobamoviruses.

Infectious clone of plant viruses provides a useful tool for studying the function of viral proteins and the interactions between virus and host. Infectivity experiments demonstrated that the infectious clone of TrMMV constructed in this study could form biologically active rod-shaped viral particles *in vivo* and were able to infect *N. benthamiana, T. kirilowii*, and other five tested cucurbitaceous crops, indicating that TrMMV could recruit similar host factors of these species to establish a successful infection. Compared with the mild mosaic and mottling symptoms of *T. kirilowii*, TrMMV infection on the other five cucurbitaceous crops produced more obvious symptoms, suggesting that TrMMV could be a potential threat to a wide range of cucurbit crops.

Tobamoviruses have extremely stable virus particles and accumulate to high titer in susceptible host plants. They are easily transmitted by mechanical means. Some tobamoviruses such as tobacco mosaic virus, cucumber green mottle mosaic virus, and tomato brown rugose fruit virus have posed a serious threat to agriculture (Lartey et al., 1996; Dombrovsky et al., 2017; Jones, 2021). Although most of the tobamovirus species are transmitted by seeds to a low percentage, the low occurrence of virus-contaminated seeds is sufficient to initiate a primary infection and facilitates virus spread to different parts of the world. In this study, we provide evidence that TrMMV could be mechanically transmitted to its natural host, *N. benthamiana*, and other five tested cucurbit plants. Although it is not known whether TrMMV is seed-borne at this stage, the availability of the infectious clone developed here would allow us to address this issue in the near future.

## Data Availability Statement

The datasets presented in this study can be found in online repositories. The names of the repository/repositories and accession number(s) can be found in the article/Supplementary Material.

## Author Contributions

XY and XZ conceived and designed the research. CC, MD, DP, WL, and JX performed the experiments. CC and XY analyzed the data. CC, XY, and XZ wrote and reviewed the manuscript. All authors have read and agreed to the published version of the manuscript.

## Conflict of Interest

The authors declare that the research was conducted in the absence of any commercial or financial relationships that could be construed as a potential conflict of interest.

## Publisher’s Note

All claims expressed in this article are solely those of the authors and do not necessarily represent those of their affiliated organizations, or those of the publisher, the editors and the reviewers. Any product that may be evaluated in this article, or claim that may be made by its manufacturer, is not guaranteed or endorsed by the publisher.
