# Supplementary material for: A Distinct Tobamovirus Associated With Trichosanthes kirilowii Mottle Mosaic Disease
Source: Front Microbiol. 2022 Jun 21;13:927230. doi: 10.3389/fmicb.2022.927230 (PMC9253623; doi:10.3389/fmicb.2022.927230)
Supplement: Supplementary file 1 [file Data_Sheet_1.PDF]

FIGURE S1. Phylogenetic trees constructed using the amino acid sequences of ORF1 **(A)**, ORF2 **(B)**, ORF3 (**C**), and ORF4 **(D)** of members in the genus *Tobamovirus*. Bootstrap values (%) for 1000 replicates are indicated. Virus names are shown in Table S2.

FIGURE S1

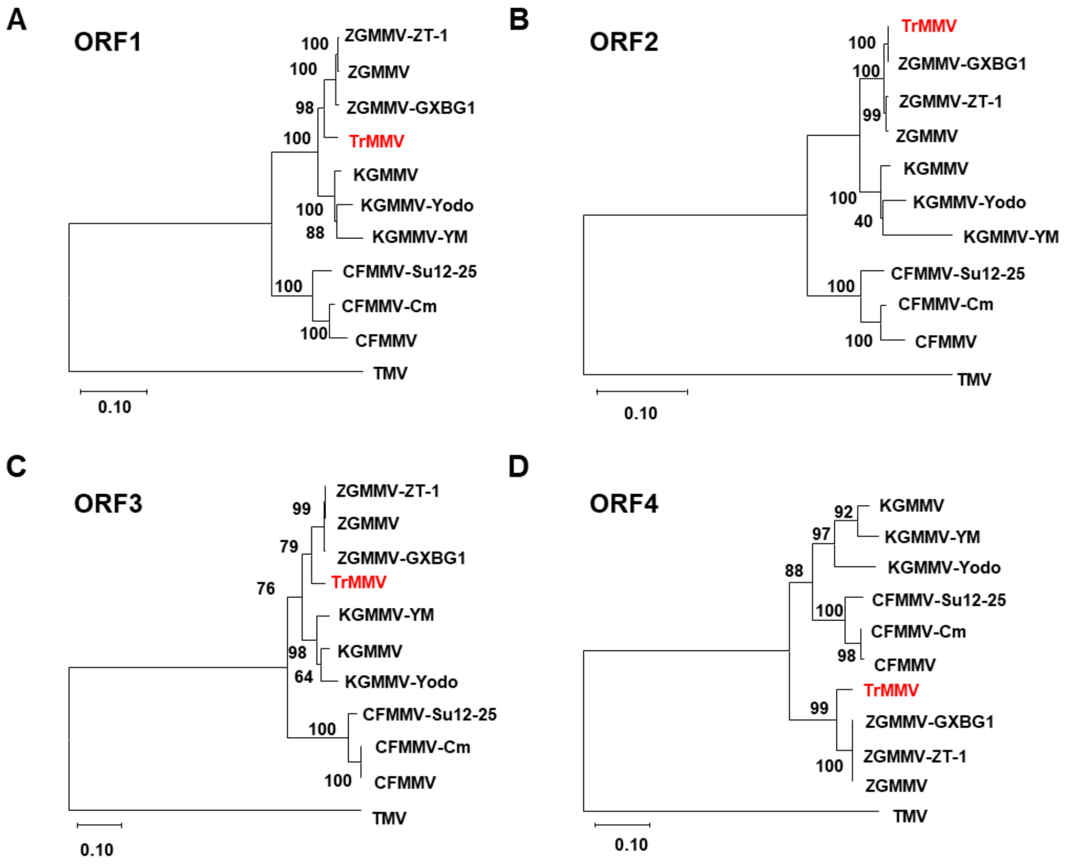
A
ORF1
ZGMMV-ZT-1
ZGMMV
ZGMMV-GXBG1
TrMMV
KGMMV
KGMMV-Yodo
KGMMV-YM
CFMMV-Su12-25
CFMMV-Cm
CFMMV
TMV
100
100
98
100
100
88
100
100
0.10
B
ORF2
TrMMV
ZGMMV-GXBG1
ZGMMV-ZT-1
ZGMMV
KGMMV
KGMMV-Yodo
KGMMV-YM
CFMMV-Su12-25
CFMMV-Cm
CFMMV
TMV
100
100
99
100
40
100
100
0.10
C
ORF3
ZGMMV-ZT-1
ZGMMV
ZGMMV-GXBG1
TrMMV
KGMMV-YM
KGMMV
KGMMV-Yodo
CFMMV-Su12-25
CFMMV-Cm
CFMMV
TMV
99
79
76
98
64
100
100
0.10
D
ORF4
KGMMV
KGMMV-YM
KGMMV-Yodo
CFMMV-Su12-25
CFMMV-Cm
CFMMV
TrMMV
ZGMMV-GXBG1
ZGMMV-ZT-1
ZGMMV
TMV
92
97
88
100
98
99
100
0.10
